# Chemical probing of RNA with the hydroxyl radical at single-atom resolution

**DOI:** 10.1093/nar/gku934

**Published:** 2014-10-13

**Authors:** Shakti Ingle, Robert N. Azad, Swapan S. Jain, Thomas D. Tullius

**Affiliations:** 1 Department of Chemistry, Boston University, Boston, MA 02215, USA; 2 Program in Bioinformatics, Boston University, Boston, MA 02215, USA

## Abstract

While hydroxyl radical cleavage is widely used to map RNA tertiary structure, lack of mechanistic understanding of strand break formation limits the degree of structural insight that can be obtained from this experiment. Here, we determine how individual ribose hydrogens of sarcin/ricin loop RNA participate in strand cleavage. We find that substituting deuterium for hydrogen at a ribose 5′-carbon produces a kinetic isotope effect on cleavage; the major cleavage product is an RNA strand terminated by a 5′-aldehyde. We conclude that hydroxyl radical abstracts a 5′-hydrogen atom, leading to RNA strand cleavage. We used this approach to obtain structural information for a GUA base triple, a common tertiary structural feature of RNA. Cleavage at U exhibits a large 5′ deuterium kinetic isotope effect, a potential signature of a base triple. Others had noted a ribose-phosphate hydrogen bond involving the G 2′-OH and the U phosphate of the GUA triple, and suggested that this hydrogen bond contributes to backbone rigidity. Substituting deoxyguanosine for G, to eliminate this hydrogen bond, results in a substantial decrease in cleavage at G and U of the triple. We conclude that this hydrogen bond is a linchpin of backbone structure around the triple.

## INTRODUCTION

To understand the myriad roles of RNA in biology, it will be necessary to define the three-dimensional structures of a large (and growing) collection of RNA molecules (1). A particular challenge is determining the structure of an RNA molecule in solution. One promising approach uses chemical probe experiments to map RNA secondary and tertiary structure (2,3). Chemical probe data are then used to drive computational prediction of the structure of the RNA (4,5). In one version of this approach, RNA secondary structure is mapped by the SHAPE method (6,7), which distinguishes base-paired from loop regions by the degree of acylation of ribose 2′-OH groups. The tertiary fold of the RNA molecule is revealed by the pattern of strand breaks produced by the hydroxyl radical, which maps the solvent exposure of the nucleic acid backbone (3,8,9).

Interpretation of the SHAPE experiment has benefited from extensive mechanistic investigations (10) that have provided precise definition of the ribonucleoside conformations that give rise to high SHAPE reactivity. In contrast, the level of structural insight available from the hydroxyl radical cleavage experiment has been constrained by our limited knowledge of the chemical details of RNA cleavage by the hydroxyl radical.

**Figure 1.**
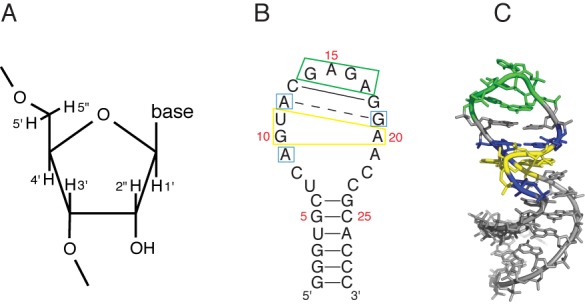
Sequence and structure of the sarcin/ricin loop (SRL) RNA molecule. (**A**) Numbering system for ribose hydrogen atoms. (**B**) Sequence and secondary structure of the SRL. Solid lines, Watson–Crick base pairs; broken line, sheared A-G base pair. Important features of the SRL are boxed: yellow, GUA base triple; blue, conserved residues flanking the GUA base triple; green, GNRA tetraloop. Nucleotides are numbered as referred to in the text. (**C**) Three-dimensional structure of the SRL (PDBID 1Q9A). Blue, yellow and green residues correspond to the color scheme in (B).

The hydroxyl radical produces a strand break in a nucleic acid molecule by abstracting a hydrogen atom from the sugar-phosphate backbone (11). There are hydrogen atoms attached to each of the five ribose carbons (Figure 1A), so in principle the hydroxyl radical could abstract any of these hydrogens. To interpret the hydroxyl radical cleavage experiment, it is presumed that the solvent accessibility of a hydrogen atom modulates its reactivity (8,9,12), thereby yielding a map of solvent accessible surface area that is based on the relative extent of cleavage at each nucleotide of an RNA. So, while the structural resolution of the experiment is conventionally defined by how often a nucleotide is cleaved, the chemistry underlying the method actually interrogates the accessibilities of single hydrogen atoms (11,13). If we could weigh the contributions of individual hydrogen atoms to the cleavage pattern, the resolution of this chemical probe experiment would reach the single-atom level.

Here we use two complementary experimental approaches to measure the reactivity of the hydroxyl radical with each of the ribose carbon–hydrogen atoms of the sarcin/ricin loop (SRL), a model RNA molecule (14). We first performed hydroxyl radical cleavage experiments on a set of specifically deuterated SRL RNA molecules. We found that deuteration of the ribose 5′-carbon leads to a large decrease in cleavage, because of the deuterium kinetic isotope effect (KIE). This is strong evidence that abstraction of a hydrogen atom from the ribose 5′-carbon leads to strand cleavage. We next discovered that two chemically distinct 5′ termini are produced at a hydroxyl radical-induced strand break. We assigned the terminus that is produced in the highest yield, a 5′-aldehyde moiety, as the product of 5′-hydrogen abstraction by the hydroxyl radical. Additional experiments provided information on the relative reactivities of the other ribose hydrogen atoms. We then used this approach to investigate the structural consequences of eliminating a single backbone hydrogen bond from a GUA base triple, the characteristic tertiary structural element of the SRL, to illustrate the level of detail that can be obtained from the hydroxyl radical cleavage experiment.

## MATERIALS AND METHODS

### Synthesis of deuterated nucleoside triphosphates (NTPs)

Strategies for isotopic labeling of RNA for structural studies have been reviewed (15). Five specifically deuterated riboses (D-[1′-^2^H]-ribose, D-[2″-^2^H]-ribose, D-[3′-^2^H]-ribose, D-[4′-^2^H]-ribose and D-[5′,5″-^2^H_2_]-ribose) were purchased from Omicronbio, Inc. (South Bend, IN, USA) and were used without further purification. Deuterium incorporation in each was greater than 98%, as indicated by the manufacturer. We synthesized specifically deuterated ribonucleoside triphosphates (A, G, C, U) (16) by adapting the enzymatic method introduced by Tolbert and Williamson (17). Briefly, the one-pot synthesis of a deuterated ribonucleoside triphosphate was accomplished by mixing a specifically deuterated ribose with a nucleobase (A, G or U) and a cocktail of enzymes. We transformed deuterated uridine triphosphate (UTP) into deuterated cytidine triphosphate (CTP) by reaction with CTP synthetase. Reaction progress was monitored by high performance liquid chromatography. Deuterated NTPs were purified by column chromatography using a DEAE anion exchange resin and characterized by ^1^H-NMR and mass spectrometry.

### Preparation of RNA molecules

RNA molecules were prepared by *in vitro* transcription using T7 RNA polymerase (Life Technologies, Grand Island, NY, USA). The RNA molecules synthesized were 5′-GGGUGCUCAGUACGAGAGGAACCGCACCC-3′, a 29-mer RNA containing the sequence of the rat sarcin/ricin loop, and 5′-GGCCUGAGUAAGCGGCGAGCGAAAGGGCC-3′, a 29-mer RNA containing the sequence of Helix 13 from *Thermus thermophilus* 23S ribosomal RNA.

To synthesize an RNA molecule, a DNA strand coding for the SRL (5′-GGGTGCGGTTCCTCTCGTACTGAGCACCCTATAGTGAGTCGTATTA-3′), or ribosome Helix 13 (5′-GGCCCTTTCGCTCGCCGCTTACTCAGGCCTATAGTGAGTCGTATTA-3′), was annealed with a DNA strand containing the T7 promoter (5′-TAATACGACTCACTATAGG-3′) to generate a partial duplex for use in a run-off transcription reaction (18). Partial duplexes were prepared in buffer (10-mM Tris-ethylenediaminetetraacetic acid (EDTA), 50-mM NaCl, pH 8.0) at a DNA template concentration of 1 mM. For synthesis of non-deuterated RNAs, a transcription reaction contained 100-μM DNA template, 1 μl each of the four NTPs (at 75-mM concentration) and 2 μl of enzyme mix from the Megashortscript T7 RNA polymerase kit (Life Technologies) per 20-μl transcription reaction. To synthesize a deuterated RNA molecule, a specifically deuterated ribonucleoside triphosphate was substituted for one of the standard NTPs. Transcription was carried out at 25°C for 2 h. At the end of the reaction, the DNA template was degraded by Turbo-DNase (Life Technologies), enzymes were removed by phenol/chloroform extraction and the RNA transcript was separated from excess NTPs using a NucAway spin column (Life Technologies).

SRL RNA with deoxyguanosine incorporated at position 10, which we refer to as deoxyG10-SRL, was purchased from IDT (Coralville, IA, USA) and used without further purification.

### RNA radiolabeling: 5′-end labeling

RNA was dephosphorylated by treatment with Antarctic Phosphatase (New England Biolabs, Ipswich, MA, USA) and 5′-end labeled with [γ-^32^P]ATP (3000 Ci/mmol) (Perkin Elmer, Waltham, MA, USA) using T4 polynucleotide kinase (New England Biolabs). Enzymes were removed by phenol/chloroform extraction. Unincorporated [γ-^32^P]ATP was separated from radiolabeled RNA using a NucAway spin column.

### RNA radiolabeling: 3′-end labeling

RNA (10–20 pmol), 3-μl 10X T4 RNA ligase I buffer, 3-μl dimethyl sulfoxide (DMSO), 0.5-μl adenosine triphosphate (ATP) (75 mM), 2-μl T4 RNA ligase I (New England Biolabs) and 5-μl cytidine-3′,5′-bis(phosphate), [5′-^32^P] (pCp, [5′-^32^P]) (Perkin Elmer) were mixed to a final volume of 30 μl in an Eppendorf tube. The ligation reaction was carried out overnight at 4°C. The enzyme was removed by phenol/chloroform extraction. Labeled RNA was separated from unincorporated pCp, [5′-^32^P] by passage through a NucAway spin column. Note that an additional nucleotide is added to the 3′ end of the RNA molecule in this labeling reaction, so the length of 3′-radiolabeled SRL RNA is 30 nt.

### Purification of radiolabeled RNA

Radiolabeled RNA was electrophoresed on a 10% denaturing polyacrylamide gel, extracted from a gel slice using the crush and soak technique, and ethanol precipitated. Radiolabeled RNA was dissolved in buffer (10-mM phosphate (pH 7.6), 10-mM NaCl, 50-mM KCl and 1-mM EDTA) and renatured by heating at 90°C for 2 min followed by slow cooling to room temperature.

### Hydroxyl radical cleavage

Radiolabeled RNA (7 μl, 20–50 kcpm) was placed in a 1.7-ml Eppendorf microcentrifuge tube. Separate 1-μl drops of the three freshly prepared Fenton reagents (1 mM [Fe(EDTA)]^2−^, 0.6% H_2_O_2_ and 10-mM sodium ascorbate) were placed on the inside wall of the reaction tube and then mixed with the RNA sample by gentle tapping. The reaction was allowed to proceed at room temperature for 1 min and then quenched by addition of 1-μl 100-mM thiourea.

Cleavage products were ethanol precipitated, heated to 90°C for 2 min, loaded onto a 20% denaturing polyacrylamide gel and electrophoresed for 3.5 h at 60 W constant power.

For 3′-radiolabeled RNA that had been cleaved by hydroxyl radical and then treated with sodium borohydride (see below), cleavage products were not heated at 90°C prior to loading on a denaturing polyacrylamide gel, because we noted that heating led to a decrease in the amount of 5′-aldehyde-terminated fragments with a concomitant increase in the amount of 5′-phosphate-terminated fragments. This behavior is analogous to the **β-**elimination of a 5′-aldehyde terminus in a DNA molecule to produce a 5′-phosphate terminus, a free base and furfural (13).

Following electrophoresis, the gel was dried and then exposed to a phosphor screen for 18 h. Gel bands were imaged using a Typhoon Trio+ Imager (GE).

### Sodium borohydride reduction

After gel purification, 3′-radiolabeled RNA was subjected to hydroxyl radical cleavage as described above. To the quenched reaction mixture was added 1 μl of freshly prepared 1-M NaBH_4_ in buffer (50-mM potassium phosphate (pH 7.6), 100-mM NaCl and 500-mM KCl). After 2 min the reaction was stopped by addition of 3.5 v/v 100% ethanol and 1-μg carrier tRNA, and the products were precipitated.

### Measurement of the deuterium KIE

Quantitation and analysis of electrophoretic band intensities was carried out using the Semi Automated Footprinting Analysis (SAFA) program (19). We evaluated the apparent KIE by calculating the ratio of the integral of a band in the control (non-deuterated) RNA lane and the corresponding band integral in the deuterated RNA lane. This ratio is equivalent to a measurement of *k*_H_/*k*_D_, the ratio of the reaction rates for a normal versus a deuterated sample, which is defined as the deuterium KIE (20). Band integrals for non-deuterated positions gave ratios of 1.0 ± 0.1, yielding a set of internal standards by which to ensure the quality of isotope effect data. Deuterium KIEs are reported as the average of triplicate experiments.

### Calculation of solvent-accessible surface areas

The solvent accessible surface areas of individual ribose hydrogen atoms in the SRL RNA molecule were calculated using the program naccess (http://www.bioinf.manchester.ac.uk/naccess/) with a probe radius of 1.4 Å, corresponding to the radius of a water molecule. The reported values represent the average surface area computed from a set of 11 crystal structures of the SRL (see Supplementary Table S1). We used the PyMOL Molecular Graphics System (Version 1.5.0.4, Schrödinger, LLC) to produce images of the SRL and Helix 13.

## RESULTS

### The SRL RNA molecule

For our experiments we used a 29-mer oligoribonucleotide that contains the sequence of the structurally well-defined SRL from rat 28S ribosomal RNA (14,21,22). We were attracted to the SRL by the variety of structural motifs and elements, including a bulged G motif (23) and a GAGA tetraloop (24), that are present within only 29 nt. In Figure 1B and C, we depict the secondary and three-dimensional structure of the SRL. The bulged G (G10) extends into the major groove and takes part in a base triple interaction with the reverse Hoogsteen base pair U11-A20. The backbone surrounding the bulged G forms an S-turn (22,25). A sharp kink occurs in the strand opposite the S-turn (25), leading to low solvent accessibility of residue A20. Other characteristic features of the bulged G motif include cross-strand stacks between A12 and A20 and between G10 and G19. A canonical C-G base pair (C13-G18) separates the bulged G motif from a GAGA tetraloop, a member of the commonly observed GNRA tetraloop family (where N is any nucleotide and R is purine) (24,26).

### Deuterium KIEs on hydroxyl radical cleavage

To determine which ribose hydrogen atoms are abstracted by the hydroxyl radical, we performed a series of deuterium KIE experiments (9,20). We began by synthesizing a set of 20 ribonucleoside triphosphates, in which each ribonucleotide (ATP, UTP, CTP or GTP) contained one of the five specifically deuterated riboses (either 1′-D, 2″-D, 3′-D, 4′-D or 5′,5″-D_2_) (16,17). Using *in vitro* transcription we prepared 20 different specifically deuterated SRL RNA molecules. Each of the 20 deuterated SRLs had one of the four nucleotides (G, for example) specifically deuterated at one of the ribose carbons (for example, the 4′-carbon). The other three nucleotides were not deuterated. Each deuterated SRL was radiolabeled and subjected to hydroxyl radical cleavage. We compared the cleavage pattern of a deuterated SRL to the cleavage pattern of a control SRL RNA prepared with all natural (non-deuterated) nucleotides.

**Figure 2.**
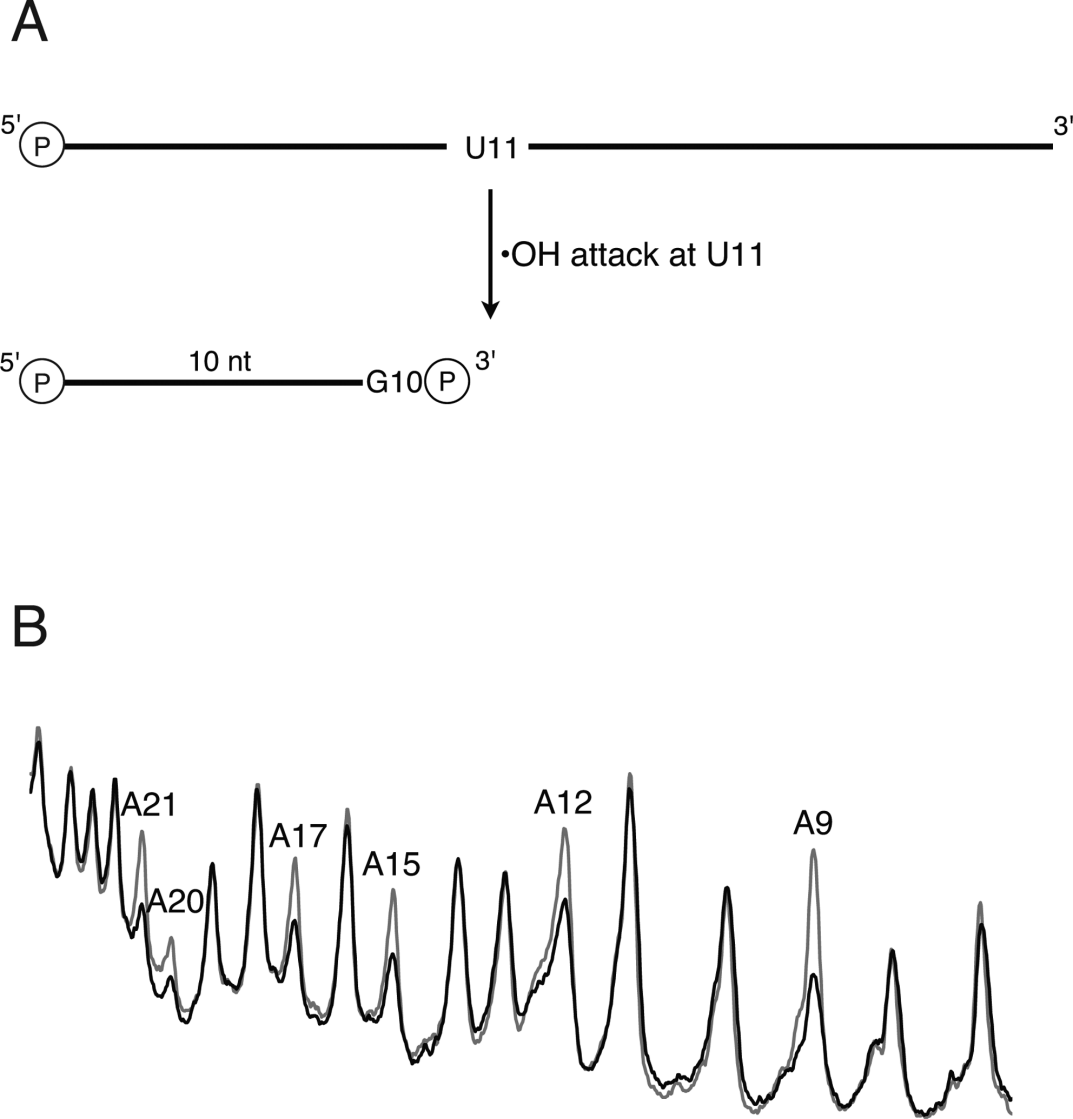
Deuterium KIE on hydroxyl radical cleavage of the SRL. (**A**) For 5′-radiolabeled RNA, the major cleavage product is a strand, terminated by 3′-phosphate, that is missing the nucleotide that was attacked by the hydroxyl radical. Gel bands are therefore identified by the nucleotide that was attacked, and not by the nucleotide at the strand terminus. (**B**) Shown are overlaid scans of gel lanes in which were separated cleavage products of SRL containing all natural nucleotides (gray), and SRL in which [5′,5″-^2^H_2_]-adenosine had been incorporated (black). The SRL was radiolabeled at the 5′ end. Major peaks represent cleavage products terminated by 3′-phosphate. Substantial decreases in cleavage upon deuteration occur only for products of cleavage at adenine residues.

Supplementary Figure S1 shows the results of a representative cleavage experiment performed on 5′-radiolabeled SRL RNA in which all adenines were dideuterated at the 5′-carbon. We assign each prominent band on the gel as a 3′-phosphate-terminated fragment that lacks the nucleotide that was attacked by the hydroxyl radical (Figure 2A). In Figure 2B, we compare scans of gel lanes containing the cleavage products of non-deuterated SRL and SRL in which all adenines were 5′-dideuterated. We observe a substantial decrease in cleavage of 5′-dideuterated adenines compared to cleavage of adenines in the control sample. For each of the adenines, the ratio of the integral of the gel band for that nucleotide in the control compared to the deuterated sample was greater than 1.0. We interpret the lower reactivity of the deuterated nucleotides as a deuterium KIE on hydroxyl radical cleavage (i.e. equivalent to a measurement of *k*_H_/*k*_D_) (20). For non-deuterated nucleotides (G, C, U) in the same sample the ratio is very close to 1.0, as expected.

We carried out similar hydroxyl radical cleavage experiments on SRL molecules containing the other 19 deuterated nucleotides (Supplementary Figures S2–S6). We then calculated KIEs for residues U7-G24 of the SRL. 5′ KIE values (Supplementary Figure S2) ranged (except for residue U11) from 1.19 to 1.65. The 5′ KIE value for U11, 1.96, was substantially greater. 4′ KIE values (Supplementary Figure S3) ranged from 1.0 to 1.21. We found that substitution of a deuterium atom at the 3′, 2′ or 1′ ribose position resulted in an apparent KIE near 1.0 for production of the 3′-phosphate-terminated product that is assigned to cleavage at that residue (Supplementary Figures S4–S6). We conclude from these experiments that hydrogen atom abstraction from the 5′ and 4′ carbons of ribose constitutes the predominant mode of reactivity of the hydroxyl radical with RNA.

### An unusually large 5′ KIE is the signature of a GUA base triple

Recall that the deuterium KIE is much larger in magnitude for the 5′-dideuterated U11 residue of the SRL compared to any other KIE that we measured (Supplementary Figure S2). Since U11 takes part in the GUA base triple motif, we asked whether an unusually large 5′ KIE is characteristic of base triples in other RNA molecules. We chose to study Helix 13 from 23S rRNA. Similar to the SRL, Helix 13 contains a GUA base triple, an S-turn and a bulged G motif (Supplementary Figure S7A and B). Although the GUA base triple in Helix 13 is capped by a larger loop than is the SRL, the overall folded structure is very similar. We prepared the 5′-dideuterated-U analog of Helix 13, radiolabeled the RNA molecule at the 5′ end, subjected the deuterated RNA to hydroxyl radical cleavage and measured the deuterium KIE (Supplementary Figure S7C and D). We found that the U residue of the GUA base triple in Helix 13 exhibits a 5′ KIE that is unusually large in magnitude (1.90) and comparable to the magnitude to the 5′ KIE of residue U11 in the SRL. We performed a similar experiment in which 5′-dideuterated-G was incorporated in Helix 13 (Figure S7E), and found no KIE greater than 1.5, again similar to our results for the SRL (Supplementary Figure S2). We conclude that the structural environment that exists in a GUA base triple leads to unusual reactivity of the hydroxyl radical toward the 5′-hydrogens of the U residue, revealed as a signature 5′ deuterium KIE for 5′-radiolabeled RNA. This large isotope effect likely results from nearly exclusive abstraction of a 5′-hydrogen atom from the U of the GUA triple (see Supplementary Data and Supplementary Figure S8).

### Detection of a 5′-aldehyde terminus at the cleavage site

We obtained further evidence that the hydroxyl radical abstracts 5′-hydrogen atoms from RNA by analyzing the strand termini produced in the cleavage reaction. In DNA, abstraction of a 5′-hydrogen atom produces a 5′-aldehyde terminus at the site of cleavage (9,11,13). A DNA strand with a 5′-aldehyde terminus migrates approximately one nucleotide slower on a denaturing gel than a strand of the same length terminated by phosphate (27). The aldehyde-terminated product therefore nearly comigrates with a phosphate-terminated strand one nucleotide longer, making it difficult to identify in a cleavage pattern. Treatment with sodium borohydride converts a 5′-aldehyde to a 5′-hydroxyl terminus. For strands roughly 20 nt or longer in length, a 5′-hydroxyl-terminated strand migrates between aldehyde- and phosphate-terminated strands of the same length (27). To observe these characteristic strand mobilities on a gel (28), the DNA strand must be radiolabeled at the 3′ end.

To investigate whether similar cleavage chemistry occurs for RNA we prepared a sample of 3′-radiolabeled SRL, subjected it to hydroxyl radical cleavage and separated the cleavage products on a denaturing gel. The cleavage pattern of this sample consists of one band for each nucleotide of the SRL (Figure S9, gray trace). Treatment of the cleavage products with sodium borohydride yielded two distinct bands per nucleotide (Figure S9, black trace). One of these bands, which comigrated with the original product, had mobility consistent with that of a strand terminated by 5′-phosphate. The second band, the product of borohydride treatment, comigrated with a 5′-hydroxyl-terminated fragment that was produced by alkaline hydrolysis (Figure S9, blue trace).

By analogy to DNA cleavage chemistry (27) we assign the 5′-hydroxyl-terminated RNA strands as the borohydride reduction products of 5′-aldehyde-terminated strands that were produced by abstraction of a 5′-hydrogen atom from RNA. Abstraction of a 4′- (or perhaps 3′-, 2″- or 1′-) hydrogen atom would yield 5′-phosphate-terminated strands (11,13).

**Figure 3.**
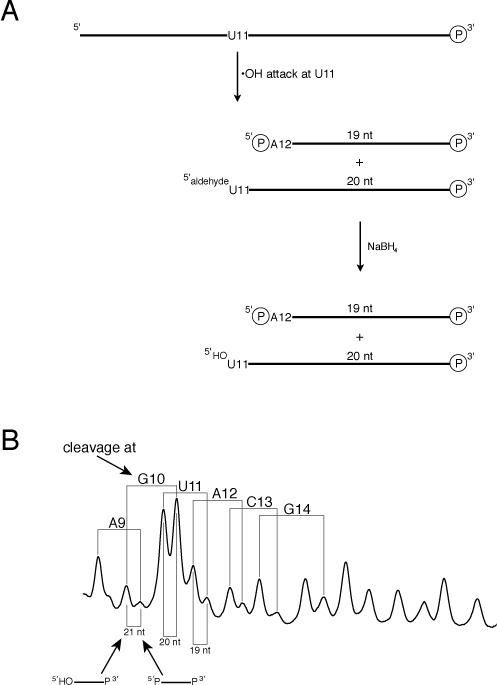
Two hydroxyl radical cleavage products are observed for each nucleotide when the SRL is 3′-radiolabeled. (**A**) For 5′-hydrogen abstraction the attacked base and sugar are still present in the product (5′-hydroxyl terminus), while for abstraction of other hydrogen atoms the base is released and the sugar is destroyed, yielding a product one nucleotide shorter (5′-phosphate terminus). Note that the lengths of RNA fragments include the extra C that is added during 3′-radiolabeling (see the Materials and Methods section). (**B**) Cleavage products were treated with sodium borohydride and electrophoresed on a denaturing gel. Assignments of representative product lengths are shown below the scan. (Note that the lengths of RNA fragments include the extra C that is added during 3′-radiolabeling.) The left-most peak in a pair corresponds to an RNA molecule terminated by 5′-hydroxyl; the right-most peak corresponds to an RNA molecule terminated by 5′-phosphate. Assignments of SRL residues at which hydrogen atom abstraction occurred are shown above the scan. For each nucleotide that was attacked, two peaks are assigned. The lower mobility fragment (left) is the product of 5′-hydrogen abstraction and is terminated by 5′-hydroxyl; the higher mobility fragment (right) is the product of abstraction of one of the other ribose hydrogens and is terminated by 5′-phosphate.

To assign each gel band to the SRL nucleotide that was attacked by the hydroxyl radical, we took into account the chemistry that results in the strand break (Figure 3A). For 5′-hydroxyl-terminated strands, the attacked nucleotide remains in the product (11,13). In contrast, 5′-phosphate-terminated strands result from initial radical abstraction of a hydrogen atom from the ribose of the 5′-adjacent nucleotide, which is followed by elimination of the phosphate and subsequent destruction of the sugar and loss of the base, and so this product is one nucleotide shorter (11,13). Thus the two bands associated with attack at a given nucleotide are not adjacent in the cleavage pattern, but instead are separated by two bands that are the products of attack at other nucleotides. The scheme for gel band assignment is depicted in Figure 3B.

We confirmed our band assignments by conducting deuterium KIE experiments on 3′-radiolabeled SRL (Supplementary Figure S10). Deuteration of the 5′-carbon of G led to a substantial decrease in band intensity for only the 5′-hydroxyl-terminated fragments that we assigned as resulting from attack at the G residues of the SRL. Similarly, we found that 4′-deuteration of G resulted in a decrease in band intensity for phosphate-terminated strands that we assigned as the products of radical attack at the guanine residues of the SRL.

It is clear from the prominence of the 5′-hydroxyl-terminated strands in the cleavage pattern of 3′-radiolabeled SRL RNA (Figure 3B) that most cleavage occurs via abstraction of a 5′-hydrogen atom. This result agrees with the greater magnitude of the observed 5′ deuterium KIEs that we discussed earlier.

We note that two bands are especially prominent in the cleavage pattern of 3′-radiolabeled SRL (Figure 3B): the 5′-phosphate-terminated strand resulting from attack at G10 and the 5′-hydroxyl-terminated strand resulting from abstraction of a 5′-hydrogen from U11. A second striking feature of the cleavage pattern is that the two bands associated with attack at G10 are reversed in relative abundance compared to all other pairs. For G10 only, the 5′-phosphate-terminated product is much more abundant than the 5′-hydroxyl-terminated product.

Because G10 and U11 are associated with the GUA base triple and the bulged-G structural motifs of the SRL, we investigated these observations in more detail.

### Structural details of the GUA base triple

**Figure 4.**
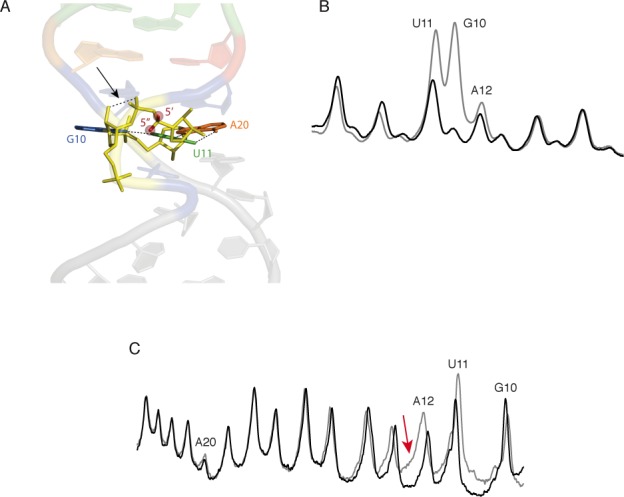
Introduction of a deoxyribose residue at position G10 of the SRL leads to profound but localized structural changes. (**A**) The GUA base triple of the SRL. Broken lines indicate hydrogen bonds in the base triple. Note in particular the hydrogen bond between the 2′-hydroxyl of G10 and the 3′-adjacent phosphate (arrow) that is discussed in the text. The 5′ and 5″ hydrogens of residue U11 that experience a large KIE when deuterated (see text) are highlighted in red. (**B**) Hydroxyl radical cleavage patterns of 3′-radiolabeled SRL (gray line) and deoxyG10 SRL (black line). (**C**) Hydroxyl radical cleavage patterns of 5′-radiolabeled SRL (gray line) and deoxyG10 SRL (black line). Red arrow: shoulder on the cleavage product assigned to residue A12 that is discussed in the text.

We now focus our attention on the GpU dinucleotide step within the GUA base triple of the SRL. It has been noted that the 5′-GpU dinucleotide platform is highly over-represented in natural RNAs (29), perhaps because of its remarkable structural rigidity. Because there is only one hydrogen bond in the G-U base pair, it was suggested that the rigidity of the GpU platform is imparted by a hydrogen bond that forms between the ribose 2′-OH of G and an oxygen atom of the phosphate that links G with U (29) (Figure 4A). The presence of this hydrogen bond in the GpU platform has been supported by detailed quantum chemical calculations (30). Having noted above that the G and U residues of the SRL GpU platform exhibit unusual reactivity toward the hydroxyl radical, we investigated whether the hydrogen bond between the 2′-OH and phosphate might be the structural basis of this reactivity.

To do this, we substituted a deoxyguanosine residue at position G10 in the SRL, to preclude formation of the 2′-OH/phosphate hydrogen bond in the GpU platform. We then performed hydroxyl radical cleavage experiments on 3′- and 5′-radiolabeled deoxyG10-SRL molecules. The cleavage pattern of 3′-radiolabeled deoxyG10-SRL (Figure 4B) was identical to that of the all-ribo SRL except for two products: the 5′-phosphate-terminated strand resulting from cleavage at G10 and the 5′-hydroxyl-terminated fragment resulting from 5′-hydrogen abstraction from U11. Both were produced in substantially lower yield for deoxyG10-SRL.

For 5′-radiolabeled deoxyG10-SRL (Figure 4C) we also observed a substantial decrease in yield for only two cleavage products: one was the fragment assigned as resulting from radical attack at U11 and the other was the fragment previously assigned as resulting from attack at A12. While a decrease in the U11 product agreed with the results of the experiment on 3′-radiolabeled deoxyG10-SRL, we were surprised that cleavage at A12 (and not G10) appeared to be affected by substitution of deoxyG10 in the SRL.

To resolve this apparent contradiction, we considered the possibility that the band running at the position of the A12 product might in fact be composed of more than one cleavage fragment. Recall that the cleavage pattern of 5′-radiolabeled RNA (Figure 2B) consists of one major product for each nucleotide, a strand terminated by a 3′-phosphate that is missing the nucleoside that was initially attacked by the hydroxyl radical (31) (Figure 2A). Strands terminated by 3′-phosphate result from abstraction of a 5′- or a 4′-hydrogen atom from the RNA backbone. Abstraction of other ribose hydrogen atoms (3′, 2″ or 1′) can yield other, more complicated, products (13).

**Figure 5.**
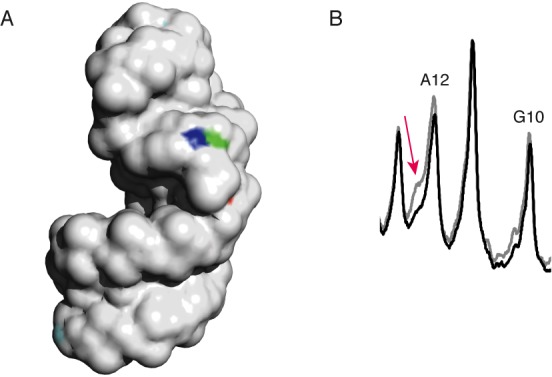
The 2″- and 3′-hydrogen atoms of residue G10 are highly solvent exposed. (**A**) Solvent-exposed surface of the SRL. The 2″-hydrogen atom of residue G10 is colored blue; the 3′-hydrogen atom of residue G10 is colored green. The 2″-hydrogen atoms of all other residues are colored cyan, and the 3′-hydrogens are colored orange. (The 2″- and 3′-hydrogens of residues other than G10 are mostly not visible in this image because their solvent accessibilities are very low.) (**B**) Overlaid scans of gel lanes in which were separated cleavage products of SRL containing all natural nucleotides (gray), and SRL in which [2″-^2^H]-guanosine had been incorporated (black). The SRL was radiolabeled at the 5′ end. While almost no difference in intensity is seen for the band assigned to cleavage at residue G10, the shoulder on the band assigned to cleavage at residue A12 decreases in intensity upon 2″-deuteration of guanine (red arrow). The mobility of this product is consistent with that of the 3′-deoxy-2′-keto-terminated strand that would result from abstraction of a 2″-ribose hydrogen atom from residue G10 (see text).

We calculated the solvent accessibilities of the ribose hydrogens of the SRL and found that the 2″- and 3′-hydrogen atoms of G10 are unusually exposed to solvent (Figure 5A). At no other nucleotide of the SRL are these hydrogens so exposed (save for those at the very ends of the molecule). What would be the product of 2″-H or 3′-H abstraction from residue G10?

Greenberg and coworkers have shown that abstraction of a 2″-hydrogen atom from the ninth nucleotide of a 19-mer RNA yields a fragment that migrates two nucleotides slower than a 3′-phosphate-terminated 8-mer (32). They assigned this fragment as a 9-mer terminated at the 3′ end with a 3′-deoxy-2′-keto moiety. If a 2″-hydrogen atom were abstracted from G10 of the SRL, we expect that a similar product, comigrating with a phosphate-terminated 11-mer, would be produced. (A 3′-phosphate-terminated 11-mer is the product of cleavage at residue A12 (Figure 2).) We had noticed in cleavage experiments on 5′-radiolabeled SRL that the band assigned as resulting from cleavage at A12 had a prominent shoulder on the low-mobility side of the band. We also noticed that a prominent shoulder is present in the equivalent band for Helix 13, residue A10 (see Supplementary Figure S7C). To see if this shoulder is associated with abstraction of the 2″-hydrogen of SRL residue G10, we re-examined the results of our kinetic isotope experiments. We noticed that deuterium substitution at C2′ of the guanines in the SRL led to a notable decrease in intensity of the A12 shoulder, but no change in the intensity of the band assigned to cleavage at G10 (Figure 5B). We take this result as evidence that the hydroxyl radical abstracts the 2″-hydrogen atom of residue G10.

While as far as we know there has been no previous determination of the products of 3′-hydrogen atom abstraction from RNA, it has been proposed for DNA that the product is a strand terminated by 3′-phosphoglycaldehyde and missing the attacked nucleotide (13,33). If similar chemistry occurs for residue G10 in SRL RNA, such a product would be expected to run more slowly on a gel compared to a 3′-phosphate-terminated fragment of the same length, perhaps comigrating with the U11 cleavage product. Further work will be needed to investigate this possibility.

From these experiments we conclude that the hydrogen bond between the 2′-OH of G10 and the 3′-adjacent phosphate enforces the distinctive hydroxyl radical reactivity of the G and U residues of the GUA triple. The hydroxyl radical abstracts almost exclusively a 5′-hydrogen from the U, leading to an unusually large KIE. The 2′-OH/phosphate hydrogen bond (29) stabilizes the bulged-G motif and so causes the 2″- and 3′-hydrogens of G10 to be exposed on the surface of the SRL (Figure 5A), making them potentially reactive toward hydroxyl radical abstraction. The lack of this hydrogen bond in deoxyG10-SRL abolishes the unusual reactivities at G and U of the GUA triple.

Since this hydrogen bond has been implicated in the structural stability of the GU platform of the GUA triple (29), we propose that in deoxyG10-SRL the GpU platform does not exist. Our data (Figure 4B and C) are consistent with the SRL backbone being more uniform in structure in deoxyG10-SRL, and in particular to lack the G-bulge that is a prominent structural characteristic of the native SRL (23).

### Correlation of the hydroxyl radical cleavage pattern with the solvent-accessible surface areas of SRL ribose hydrogens

**Figure 6.**
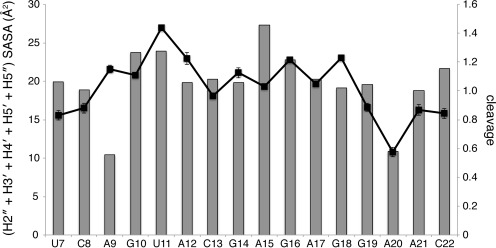
Hydroxyl radical cleavage correlates with the solvent-accessible surface area of ribose hydrogen atoms. Gray bars, sum of the solvent accessible surface areas (SASA) of the 2″-, 3′-, 4′-, 5′- and 5″-ribose hydrogen atoms; black line, cleavage (arbitrary units) with standard deviation.

Our experiments show that abstraction of a 5′-hydrogen atom is the major pathway for strand cleavage of RNA by the hydroxyl radical. How do these experimental results fit with structural features of RNA? The usual interpretation of the hydroxyl radical cleavage pattern is that the extent of cleavage at a given nucleotide corresponds to the solvent accessibility of its sugar hydrogen atoms (4,8,9,34–36). For the SRL, simply considering the solvent accessibilities of the two hydrogens attached to each ribose 5′-carbon yields a very good correlation with cleavage (Supplementary Figure S11A). On the other hand, cleavage correlates with the accessibilities of most but not all of the 4′-hydrogen atoms (Supplementary Figure S11B), because the folded structure of the SRL leads to near-zero accessibility of the 4′-hydrogens of residues A9 and G10, while the SRL backbone is still cleaved at these positions. Cleavage correlates well with the sum of the accessibilities of the 4′-, 5′- and 5″-hydrogens, except for residues A9 and G10 (Supplementary Figure S11C). Including the solvent accessibilities of the 2″- and 3′-hydrogens along with the accessibilities of the 4′-, 5′- and 5″-hydrogens (Figure 6) greatly improves the correlation with cleavage at G10 (see Figure 5A) while not affecting other residues, because 2″- and 3′-hydrogens are seldom exposed to solvent in folded RNA.

Despite the overall correspondence of cleavage and solvent accessible surface area, one SRL nucleotide, tetraloop residue A15, is cleaved much less than its calculated solvent accessibility would predict. In SRL X-ray structures (23,25) one side of the A15 base is exposed to solvent and the other is stacked on G16, ‘capping’ the GAGA tetraloop. There is evidence, though, that the capping nucleotide of a tetraloop is conformationally flexible (37–39) so its conformation in solution might not be the same as in a particular crystal structure. To investigate this question we obtained coordinates for 19 GNRA tetraloops that were identified by Williams and coworkers in ribosome X-ray structures (26) and calculated the sum of the solvent accessibilities of the ribose hydrogens for each of the tetraloop and flanking residues. We found that the standard deviation of the accessibility sum for the capping residue of the GNRA tetraloop, residue ‘N’ (equivalent to A15 in the SRL), is substantially greater than for the other tetraloop residues (Supplementary Figure S12). In most of the 19 ribosome tetraloops the capping residue adopts the stacked conformation that is seen in SRL structures. Some of the ribosome tetraloops, though, adopt conformations in which this nucleotide is not stacked. Perhaps surprisingly the solvent exposure of the ribose hydrogens of the capping nucleotide is lower in the unstacked conformations, because of the C2′-endo ribose conformation that is adopted by the unstacked nucleotide. The mixture of stacked and unstacked conformations that is found in the collection of ribosome tetraloops is the source of the variability in ribose solvent accessibility that is observed for the capping nucleotide. Our observation that there is less cleavage of the A15 capping nucleotide in the SRL than would be expected from SRL X-ray structures is consistent with this nucleotide spending some fraction of time in solution in the unstacked conformation, in which its ribose is less solvent-accessible.

Residue A9, on the other hand, is apparently cleaved substantially more than its solvent accessibility would predict (Figure 6). While we suspect that the ‘extra’ cleavage may be the result of anomalous migration of fragments resulting from hydrogen abstraction at some other residue (as we propose above for G10 and A12), we have no evidence yet that this is the case.

## DISCUSSION

We show here that the hydroxyl radical cleavage experiment can be used to quantitatively monitor the solvent accessibilities of single ribose hydrogen atoms in an RNA molecule. Single-atom resolution is achieved by taking advantage of the ability of gel electrophoresis to resolve RNA fragments of the same length that differ in the chemical nature of the strand termini (phosphate, hydroxyl, aldehyde, etc.) that are produced by the cleavage reaction (28). Further information on which hydrogen atoms are susceptible to radical attack is obtained by performing the cleavage reaction on an RNA molecule in which deuterium has replaced hydrogen at specific ribose carbons (9,20).

At nearly all nucleotides in the RNA molecule we studied, the most abundant cleavage product comes from abstraction of a hydrogen atom attached to a ribose 5′-carbon. The main evidence for this conclusion is that the cleavage product produced in highest yield is an RNA strand terminated by a 5′-aldehyde moiety (Figure 3). Additional support comes from the observation of a relatively large deuterium KIE upon cleavage of 5′,5″-dideuterated RNA (Figure 2B and Supplementary Figures S1 and S2).

Since work over several decades on free radical cleavage of DNA (11,13) has established the 5′-aldehyde terminus as the unique product of 5′-hydrogen abstraction, it is perhaps surprising that this species has not previously been reported for RNA. One reason is that mechanistic study of hydroxyl radical cleavage of RNA has lagged similar studies of DNA. The Cowan laboratory recently used MALDI-TOF mass spectrometry to obtain detailed mechanistic insight into RNA cleavage by radicals (40,41). While these workers detected several different RNA strand termini in their experiments, the 5′-aldehyde was not among them. The authors noted, though, that they used an expected-mass list of fragments to identify radical-produced strand termini. While 3′- and 5′-hydroxyl, 3′- and 5′-phosphate, 3′-phosphoglycolate and 2′,3′-cylic phosphate termini were on the list, 5′-aldehyde was not, so their experiments were not designed to detect this abundant terminus.

While most nucleotides in the relatively small RNA molecule that we studied are cleaved to about the same extent, three stand out in the cleavage pattern. It is perhaps not surprising that these nucleotides, G10, U11 and A20, form a GUA base triple, the signature structural motif of the SRL (23). In the conventional cleavage experiment, in which the RNA molecule is 5′-radiolabeled, A20 is distinguished by the lack of cleavage that occurs at this residue (Figure 2B). When the SRL is 3′-radiolabeled, G10 and U11 are identified as the most prominent sites of cleavage (Figure 3A).

An unusual feature of the GUA triple is its structural rigidity, which is thought to be conferred by a G-ribose-OH/U-phosphate hydrogen bond (Figure 4A). The distorted S-shaped backbone around the G of the triple is likely stabilized by this H-bond, facilitating the formation of a base pair between the G and the adjacent U to form a G-U platform (29). We found that the hydroxyl radical is a sensitive probe of this tertiary structural motif. We eliminated the backbone ribose-phosphate hydrogen bond by incorporating a deoxyguanosine residue at this position in the SRL sequence and found a striking, localized decrease in cleavage at the G and U residues (Figure 4B and C). The cleavage pattern becomes much more uniform, consistent with the loss of the G-U platform. We suggest that the backbone hydrogen bond in the GUA triple acts like the catch of a mousetrap, releasing the distorted backbone when disrupted. Since the bulged-G motif is a key recognition element for ribotoxins that cleave the SRL (42), this hydrogen bond is likely critical to the ability of ribotoxins to disable the ribosome.

High-throughput methods for RNA structure mapping are now in active development, to cope with the rapidly expanding universe of cellular RNAs (1,43). New enzymatic (44,45) and chemical (SHAPE) (46) methods take advantage of deep sequencing to determine the secondary structures of large collections of RNA molecules in parallel. The hydroxyl radical cleavage experiment has recently been adapted for analysis by deep sequencing for high-throughput determination of RNA tertiary structure (36). We anticipate that the mechanistic insights we present here will be useful in developing and refining new experimental approaches for RNA tertiary structure determination, including high-throughput methods.

## SUPPLEMENTARY DATA

Supplementary Data are available at NAR Online.

SUPPLEMENTARY DATA
