# Supplementary material for: Chemical probing of RNA with the hydroxyl radical at single-atom resolution
Source: Nucleic Acids Res. 2014 Oct 13;42(20):12758–67. doi: 10.1093/nar/gku934 (PMC4227780; doi:10.1093/nar/gku934)
Supplement: SUPPLEMENTARY DATA [file supp_42_20_12758__index.html]

Chemical probing of RNA with the hydroxyl radical at single-atom resolution — Chemical probing of RNA with the hydroxyl radical at single-atom resolution — SUPPLEMENTARY DATA 

# Chemical probing of RNA with the hydroxyl radical at single-atom resolution

## SUPPLEMENTARY DATA

**Files in this Data Supplement:**

- SUPPLEMENTARY DATA
