# Supplementary material for: Chemical probing of RNA with the hydroxyl radical at single-atom resolution
Source: Nucleic Acids Res. 2014 Oct 13;42(20):12758–67. doi: 10.1093/nar/gku934 (PMC4227780; doi:10.1093/nar/gku934)

Supplementary Data for

Chemical probing of RNA with the hydroxyl radical at single-atom resolution

Shakti Ingle, Robert N. Azad, Swapan S. Jain & Thomas D. Tullius

Correspondence to: tullius@bu.edu (TDT)

Interpretation of the magnitude of the deuterium kinetic isotope effect

We take the observation of a deuterium kinetic isotope effect on cleavage as evidence that a particular hydrogen atom is abstracted by the hydroxyl radical (1). (We note that the lack of observation of a kinetic isotope effect does not constitute evidence that a particular hydrogen atom is not abstracted. This is because the presence of a kinetic isotope effect requires that abstraction of the hydrogen atom must be the rate-determining step of the reaction (2). This may not always be the case.)

For the experiments we describe here, it is trickier to relate the magnitude of the isotope effect to structure, because only one prominent species (an RNA strand terminated with a 3'-phosphate) is observed upon electrophoresis of the cleavage products of 5'-radiolabeled RNA (Figure 2). For DNA, it is known that abstraction of different deoxyribose hydrogens yields the same 3'-phosphate-terminated strand break (3, 4). So, a given gel band may in principle contain cleavage products resulting from abstraction of different hydrogen atoms. This convolution of products will affect the observed value of the kinetic isotope effect.

We illustrate the problem with a simulated gel band that contains the products of two different hydrogen atom abstractions (Supplementary Figure S8). For this example we assume that the intrinsic deuterium kinetic isotope effect on hydrogen atom abstraction (k_H/k_D) is 2.6 (as we observed for DNA (1)). We consider two cases. In the first case, the integral of the observed band (blue) (in arbitrary units) is 20, and the ratio of the two products (red and green bands) is 3:1. Deuteration of the carbon atom that yields the more abundant product will cause the intensity of the (composite) blue gel band to be decreased to [(15/2.6)+5)] = 10.8. (Note that only the red band decreases in intensity upon deuteration; the intensity of the green band does not change.) The observed kinetic isotope effect therefore is k_H/k_D = 20/10.8 = 1.9. In the second case only the more abundant of the two products (the red band) is present, so the original intensity of the gel band is 15. Deuteration of the ribose carbon that gives rise to that product will decrease the band intensity to [15/2.6] = 5.8, yielding an observed deuterium kinetic isotope effect of k_H/k_D = 15/5.8 = 2.6. (This is the same as the intrinsic isotope effect, because only one product is present in the gel band.)

We conclude from this analysis that the magnitude of the deuterium kinetic isotope effect that is observed at a given nucleotide for 5'-radiolabeled RNA is related to the relative extent of hydrogen atom abstraction from the different ribose carbons of that nucleotide. If a gel band results from abstraction of a

hydrogen atom from a single ribose carbon, the observed KIE is maximal. If more than one ribose carbon atom of that residue can be abstracted by the hydroxyl radical, yielding the same product from two reaction pathways, the observed KIE is lower in magnitude.

SUPPLEMENTARY REFERENCES

1. Tullius, T.D., Balasubramanian, B. and Pogozelski, W.K. (1998) DNA strand breaking by the hydroxyl radical is governed by the accessible surface areas of the hydrogen atoms of the DNA backbone. *Proc. Natl. Acad. Sci. USA*, **95**, 9738–9743.

2. Kozarich, J.W., Worth, L., Frank, B.L., Christner, D.F., Vanderwall, D.E. and Stubbe, J. (1989) Sequence-specific isotope effects on the cleavage of DNA by bleomycin. *Science*, **245**, 1396–1399.

3. Pogozelski, W.K. and Tullius, T.D. (1998) Oxidative strand scission of nucleic acids: routes Initiated by hydrogen abstraction from the sugar moiety. *Chem. Rev.*, **98**, 1089–1108.

4. Pitié, M. and Pratviel, G. (2010) Activation of DNA carbon-hydrogen bonds by metal complexes. *Chem. Rev.*, **110**, 1018–1059.

5. Mohan, S., Hsiao, C., Bowman, J.C., Wartell, R. and Williams, L.D. (2010) RNA tetraloop folding reveals tension between backbone restraints and molecular interactions. *J. Am. Chem. Soc.*, **132**, 12679–12689.

SUPPLEMENTARY FIGURE LEGENDS

Supplementary Figure S1. Hydroxyl radical cleavage patterns of native and deuterated SRL RNA. Shown is the phosphorimage of a denaturing electrophoresis gel on which was separated the products of hydroxyl radical cleavage of 5'-radiolabeled, all-natural SRL (lanes marked H), and SRL in which [5',5"-$^{2}H_{2}$]-adenosine had been incorporated (lanes marked D). Lanes marked C, untreated SRL RNA (left, all natural nucleotides; right, deuterated nucleotides). Lanes marked T1, products of RNase T1 digestion of SRL RNA. Band assignments for the RNase T1 digestion pattern are shown at left. Arrows indicate bands resulting from hydroxyl radical cleavage at the adenines of the SRL. Note that these band assignments take into account that hydroxyl radical cleavage products lack the attacked nucleotide at the 3' end of the fragment (see Figure 2A).

Supplementary Figure S2. Deuterium kinetic isotope effect on hydroxyl radical cleavage resulting from 5',5"-dideuteration of ribose in the SRL. Left, scans of gel lanes. Grey, all natural SRL; black, 5',5"-dideuterated nucleotides were incorporated into the SRL. Right, kinetic isotope effects evaluated at each residue of the SRL. Plotted is the ratio of peak integrals for the all-natural SRL sample divided by the peak integrals of deuterated SRL. Grey bars, nucleotides that were deuterated. Error bars indicate the standard deviation for three experiments.

Supplementary Figure S3. Deuterium kinetic isotope effect on hydroxyl radical cleavage resulting from 4'-deuteration of ribose in the SRL. Left, scans of gel lanes. Grey, all natural SRL; black, 4'-deuterated nucleotides were incorporated into the SRL. Right, kinetic isotope effects evaluated at each residue of the SRL. Plotted is the ratio of peak integrals for the all-natural SRL sample divided by the peak integrals of deuterated SRL. Grey bars, nucleotides that were deuterated. Error bars indicate the standard deviation for three experiments.

Supplementary Figure S4. Effect on hydroxyl radical cleavage of 1'-deuteration of ribose in the SRL. Grey, all natural SRL; black, 1'-deuterated nucleotides were incorporated into the SRL.

Supplementary Figure S5. Effect on hydroxyl radical cleavage of 2'-deuteration of ribose in the SRL. Grey, all natural SRL; black, 2'-deuterated nucleotides were incorporated into the SRL.

Supplementary Figure S6. Effect on hydroxyl radical cleavage of 3'-deuteration of ribose in the SRL. Grey, all natural SRL; black, 3'-deuterated nucleotides were incorporated into the SRL.

Supplementary Figure S7. Ribosome Helix 13 exhibits a large deuterium kinetic isotope effect on cleavage at the U of the GUA base triple. (**A**) Sequence and secondary structure of Helix 13. Black lines,

Watson-Crick base pairs. Features of Helix 13 that correspond to the sarcin/ricin loop RNA molecule (see Figure 1B) are boxed: yellow, GUA base triple; blue, identical residues that flank the SRL and Helix 13 triples; green, Helix 13 pentaloop. (**B**) Three-dimensional structures of *E. coli* SRL (PDBID 1Q9A) and Helix 13 (1VSA, residues 235-261) superimposed. Blue, yellow and green residues correspond to the color scheme in (**A**). (**C**) Deuterium kinetic isotope effect on hydroxyl radical cleavage of Helix 13. Shown are overlaid scans of gel lanes in which were separated cleavage products of Helix 13 containing all natural nucleotides (grey), and Helix 13 in which [5',5"-$^2H_2$]-uridine had been incorporated (black). Helix 13 was radiolabeled at the 5' end. The red arrow indicates the shoulder on peak A10 that is discussed in the text. (**D**) Kinetic isotope effects evaluated at each residue of Helix 13. Plotted is the ratio of peak integrals for the all-natural Helix 13 sample divided by the peak integrals of a Helix 13 sample in which [5',5"-$^2H_2$]-uridine had been incorporated. Grey bar, nucleotide that was deuterated. Error bars indicate the standard deviation for three experiments. (**E**) Kinetic isotope effects evaluated at each residue of Helix 13. Plotted is the ratio of peak integrals for the all-natural Helix 13 sample divided by the peak integrals of a Helix 13 sample in which [5',5"-$^2H_2$]-guanosine had been incorporated. Grey bars, nucleotides that were deuterated. Error bars indicate the standard deviation for three experiments.

Supplementary Figure S8. Comigration of gel bands that result from abstraction of different ribose hydrogen atoms complicates interpretation of the observed kinetic isotope effect. Shown is a simulation of a deuterium kinetic isotope effect experiment for 5'-radiolabeled RNA. Two cases are depicted: A,B, two different ribose hydrogen atoms are abstracted by the hydroxyl radical; C,D, a single ribose hydrogen atom is abstracted. (**A**) In the first case, the band that is observed on the gel (blue) is the sum of two cleavage products that result from initial abstraction of different hydrogen atoms: red, major product; green, minor product. (**B**) Deuterium incorporation at the ribose carbon that gives rise to the major product leads to a substantial decrease in the intensity of the red band, while the intensity of the green band remains the same. The decrease in intensity of the (observed) blue band is due only to the isotope effect on production of the red product. (**C**) In the second case, only a single ribose hydrogen atom is abstracted by the hydroxyl radical, and one product (the red band) is observed. (**C**) Deuterium incorporation at the ribose carbon that gives rise to the product leads to a substantial decrease in the intensity of the red band. While the decrease in intensity of the red band is the same as in the first case (A,B), the observed kinetic isotope effect will be larger, because the observed gel bands in C and D are made up of a single product.

Supplementary Figure S9. Hydroxyl radical cleavage of 3'-radiolabeled SRL initially produces one band per nucleotide, which upon treatment with sodium borohydride is partially converted to a new band having mobility characteristic of a strand terminated by 5'-hydroxyl. Grey trace, scan of a gel lane on which was separated 3'-radiolabeled SRL RNA that had been treated with the hydroxyl radical. Black trace, scan of a gel lane on which was separated 3'-radiolabeled SRL RNA that had been treated with the hydroxyl radical

followed by treatment with sodium borohydride. Blue trace, scan of a gel lane on which was separated an alkaline hydrolysis ladder produced from 3'-radiolabeled SRL RNA, to provide a set of RNA fragments terminated by 5'-hydroxyl.

Supplementary Figure S10. The deuterium kinetic isotope effect aids in the assignment of bands in the cleavage pattern of 3'-radiolabeled SRL RNA. Subsequent to hydroxyl radical treatment, cleavage products were treated with sodium borohydride and electrophoresed on a denaturing acrylamide gel. Shown are overlaid scans of gel lanes in which were separated cleavage products of SRL RNA containing all natural nucleotides (grey), and SRL in which specifically-deuterated guanosine had been incorporated (black). (**A**) [5',5"-$^{2}H_{2}$] guanosine was incorporated in SRL RNA. A band that experiences a noticeable decrease in cleavage upon deuteration is assigned as the product of abstraction of a hydrogen atom from the 5'-carbon of guanosine followed by borohydride reduction (a 5'-hydroxyl-terminated strand (inset)). (**B**) [4'-^{2}H] guanosine was incorporated in the SRL RNA. A band that experiences a noticeable decrease in cleavage upon deuteration is assigned as the product of abstraction of a hydrogen atom from the 4'-carbon of guanosine (a 5'-phosphate-terminated strand (inset)). (Note that the intense band assigned as the 5'-phosphate-terminated product of attack at residue G10 experiences only a small decrease in intensity upon 4'-deuteration, consistent with this band being mainly the product of hydroxyl radical abstraction of other ribose hydrogens. See text for discussion.)

Supplementary Figure S11. Comparison of hydroxyl radical cleavage with the solvent-accessible surface areas of ribose hydrogen atoms. Grey bars, solvent accessible surface areas (SASA) of ribose hydrogen atoms; black line, cleavage (arbitrary units) with standard deviation. (**A**) Sum of the SASA of H5' and H5" vs. cleavage. (**B**) SASA of H4' vs. cleavage. (**C**) Sum of the SASA of H4', H5', and H5" vs. cleavage.

Supplementary Figure S12. The "capping" nucleotide of a GNRA tetraloop is conformationally flexible. We calculated the sum of the solvent-accessible surfaces areas of all ribose hydrogens for each nucleotide of 19 GNRA tetraloops that were found in 23S ribosomal RNA by Williams and coworkers (5). We plotted the mean and standard deviation (error bars) of the SASA sum for the tetraloop and four flanking residues. The capping nucleotide ("N", dark grey) has a larger mean SASA, with a substantially larger variation, compared to the other residues.

SUPPLEMENTARY TABLE

Supplementary Table S1. SRL X-ray structures used in the analyses in this paper

PDBID	Resolution (Å)	Year	Description
1Q9A	1.04	2003	Wild-type from *E. coli* 23S rRNA
1Q93	<2.25	2003	Lethal mutant (rat), C to G & G to C flanking tetraloop
1Q96	<1.75	2003	Viable mutant (rat), C to U & G to A flanking tetraloop
3DVZ	<1.00	2009	From *E. coli* 23S rRNA
3DW4	<1.00	2009	Similar to 3DVZ, U2650-OCH_3
3DW5	<1.00	2009	Similar to 3DVZ, U2656-OCH_3
3DW6	<1.00	2009	Similar to 3DVZ, U2650-$SeCH_3$
3DW7	<1.00	2009	Similar to 3DVZ, U2656-$SeCH_3$
430D	2.1	1998	From rat 28S rRNA
480D	<1.5	1999	From *E. coli* 23S rRNA
483D	1.11	1999	From *E. coli* 23S rRNA

Figure S1

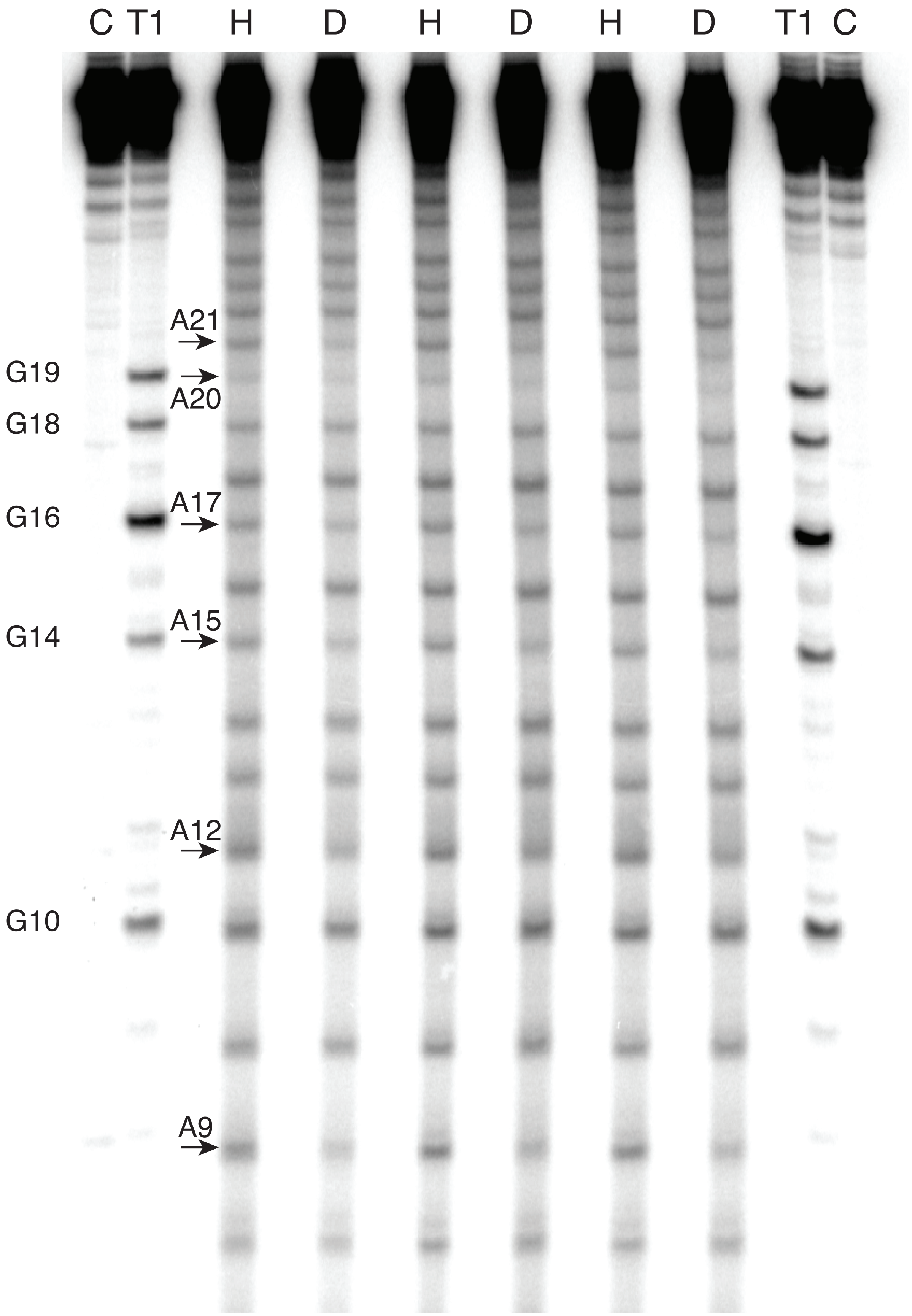

Figure S2

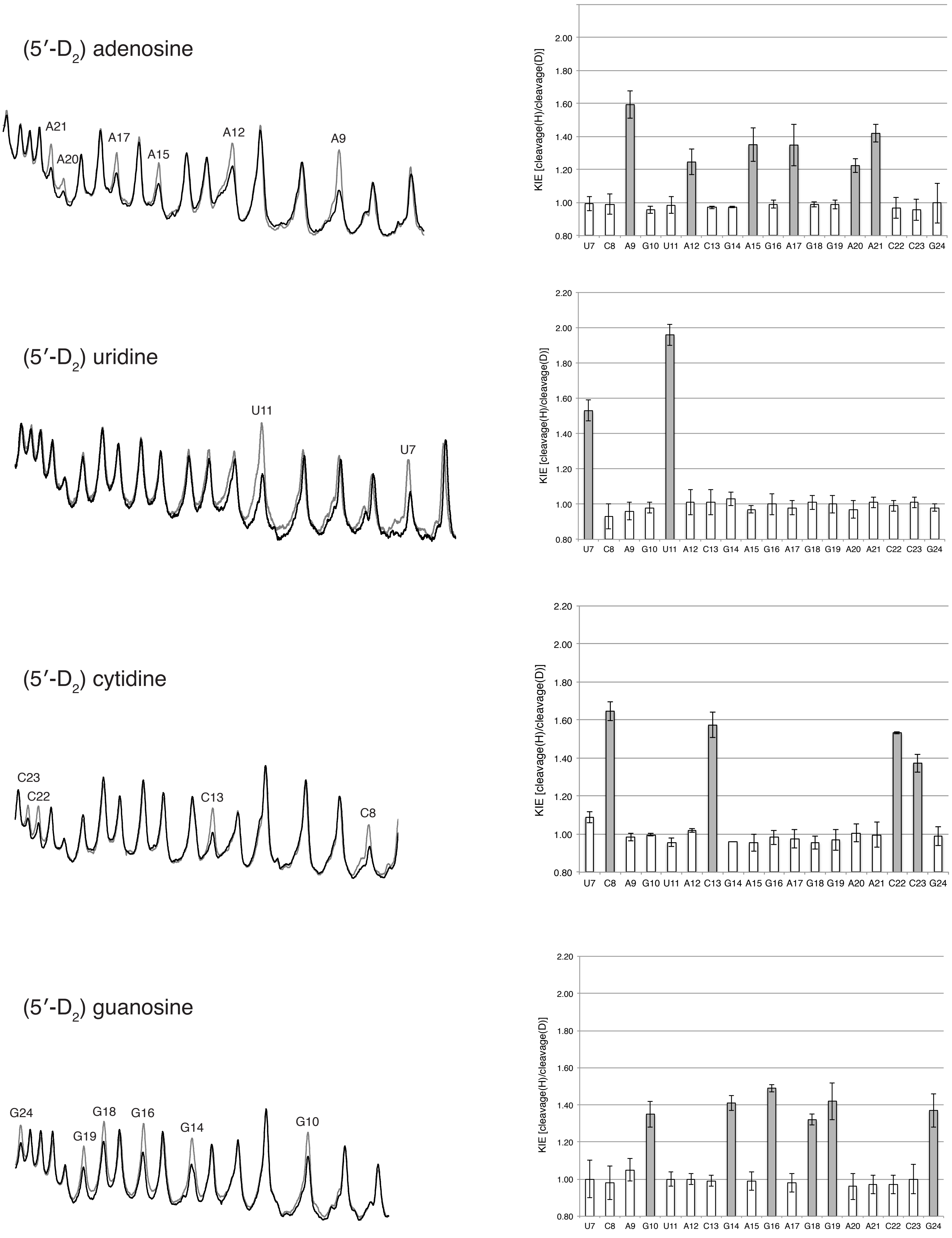

(5′-D2) adenosine
A21
A20
A17
A15
A12
A9
(5′-D2) uridine
U11
U7
(5′-D2) cytidine
C23
C22
C13
C8
(5′-D2) guanosine
G24
G19
G18
G16
G14
G10
KIE [cleavage(H)/cleavage(D)]
0.80
1.00
1.20
1.40
1.60
1.80
2.00
2.20
U7 C8 A9 G10 U11 A12 C13 G14 A15 G16 A17 G18 G19 A20 A21 C22 C23 G24

Figure S3

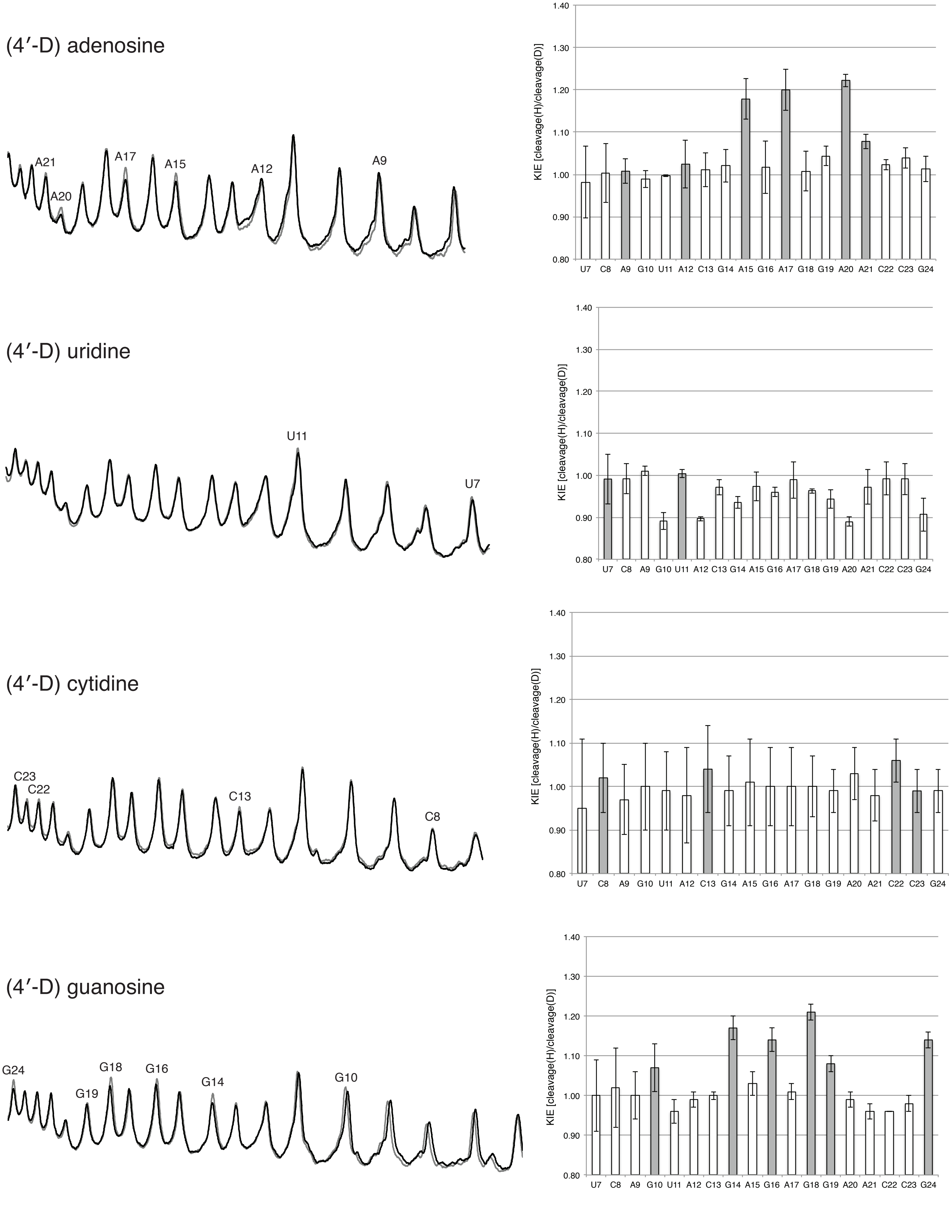

(4′-D) adenosine
A21
A20
A17
A15
A12
A9
(4′-D) uridine
U11
U7
(4′-D) cytidine
C23
C22
C13
C8
(4′-D) guanosine
G24
G19
G18
G16
G14
G10
KIE [cleavage(H)/cleavage(D)]
1.40
1.30
1.20
1.10
1.00
0.90
0.80
U7 C8 A9 G10 U11 A12 C13 G14 A15 G16 A17 G18 G19 A20 A21 C22 C23 G24

Figure S4

(1′-D) adenosine

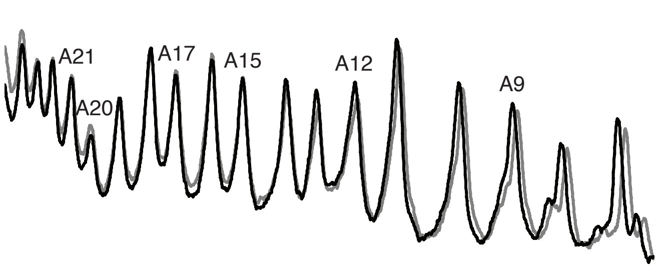

(1′-D) guanosine

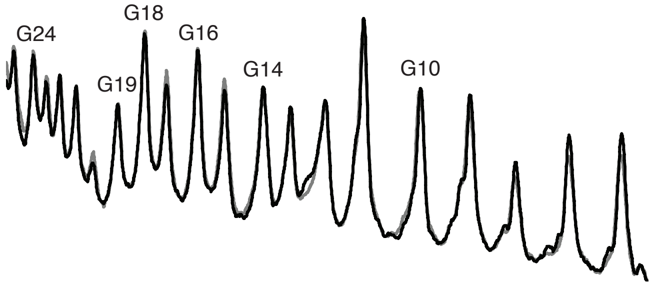

(1′-D) cytidine

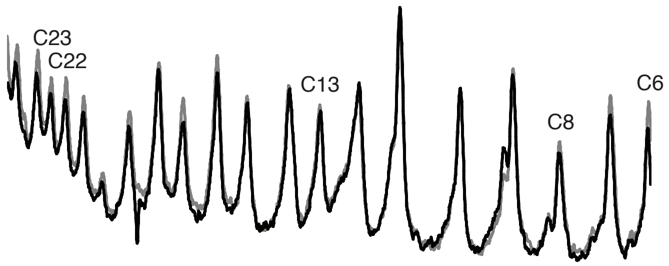

(1′-D) uridine

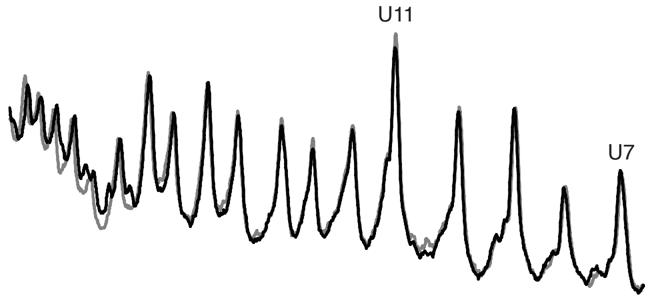

Figure S5

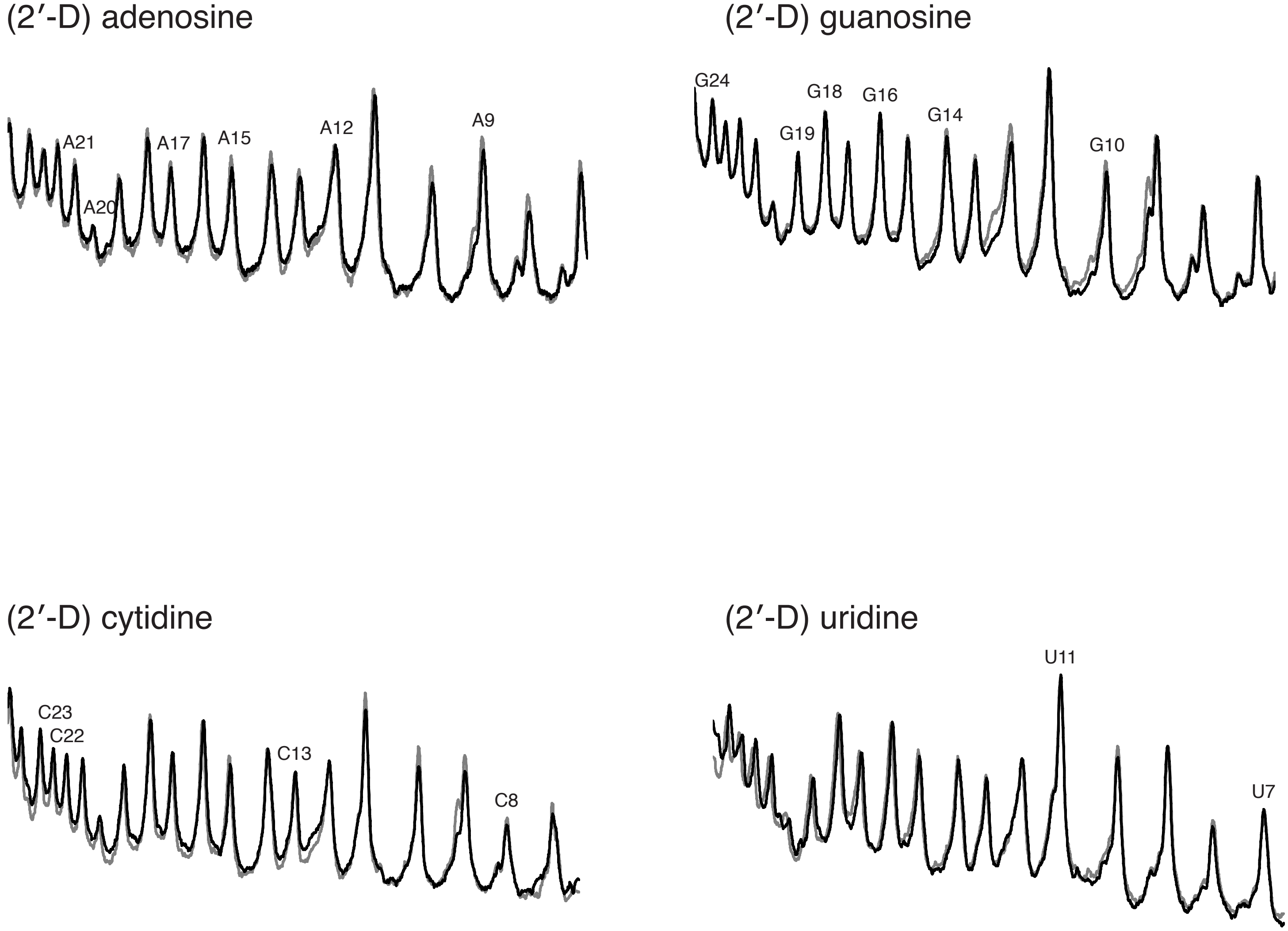
(2′-D) adenosine
A21
A20
A17
A15
A12
A9
(2′-D) guanosine
G24
G19
G18
G16
G14
G10
(2′-D) cytidine
C23
C22
C13
C8
(2′-D) uridine
U11
U7

Figure S6

(3′-D) adenosine

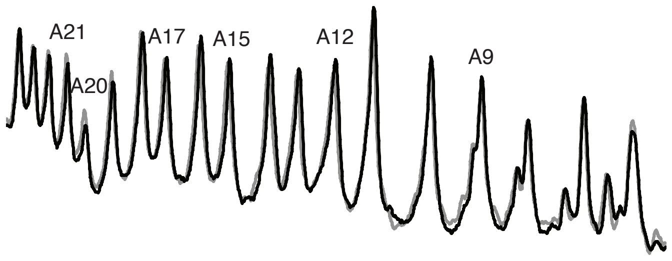

(3′-D) guanosine

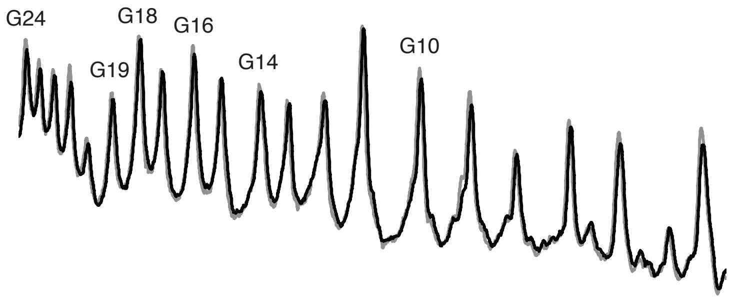

(3′-D) cytidine

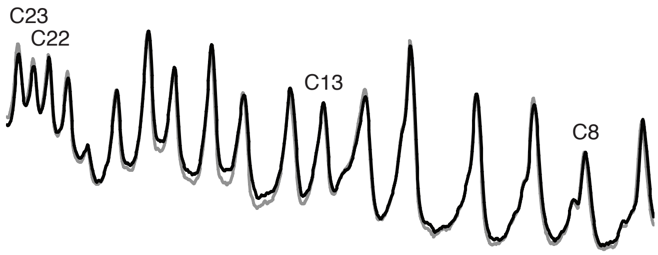

(3′-D) uridine

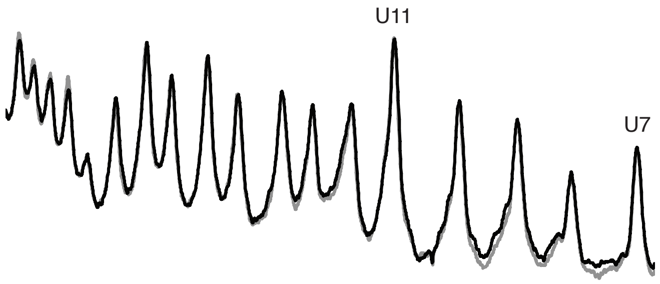

Figure S7

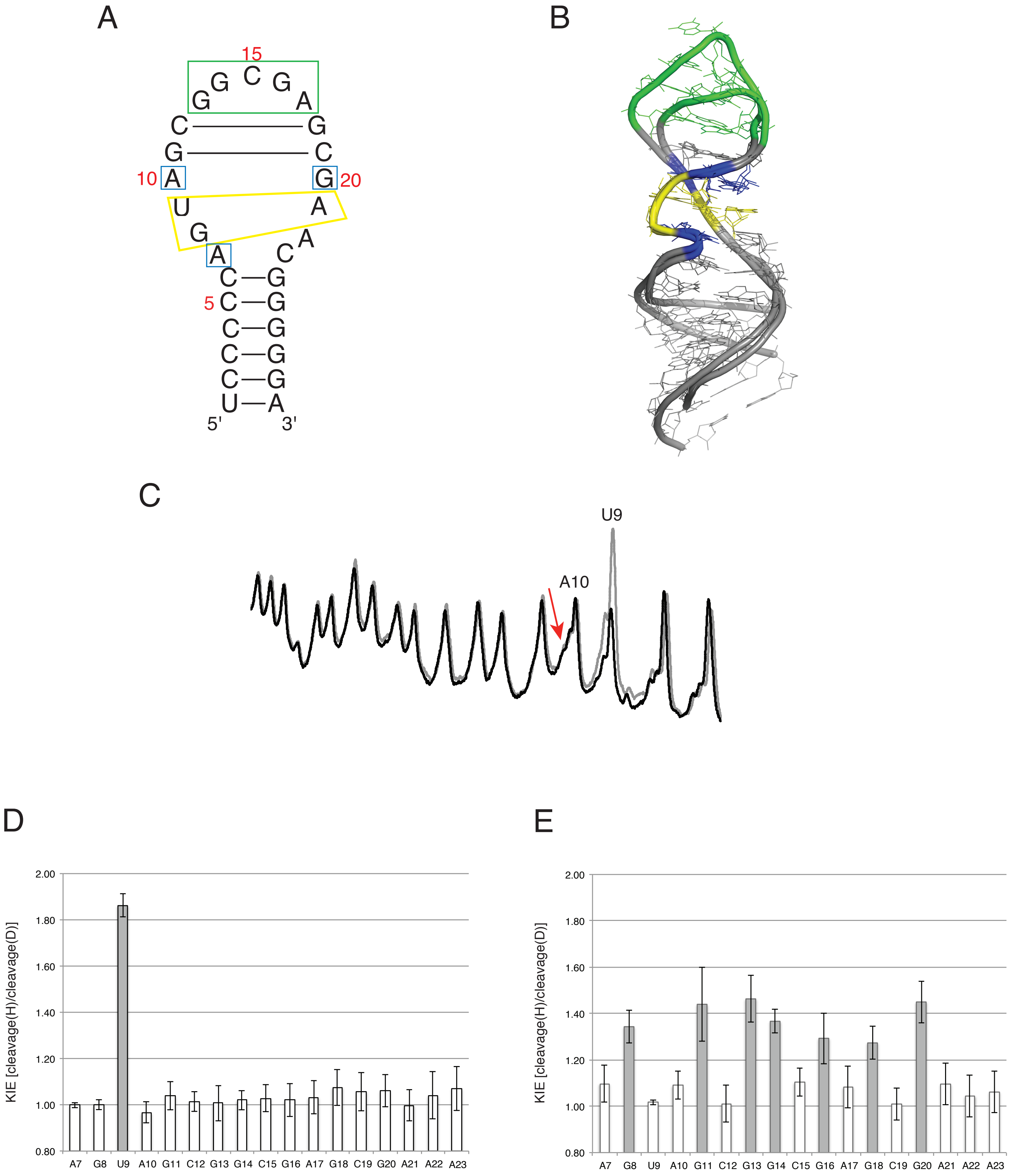
A
15
G G C G A
C G
G C
10 A G 20
U A
G A
A C
C—G
5 C—G
C—G
C—G
C—G
U—A
5'
3'
B
C
U9
A10
D
E
KIE [cleavage(H)/cleavage(D)]
2.00
1.80
1.60
1.40
1.20
1.00
0.80
A7 G8 U9 A10 G11 C12 G13 G14 C15 G16 A17 G18 C19 G20 A21 A22 A23

Figure S8

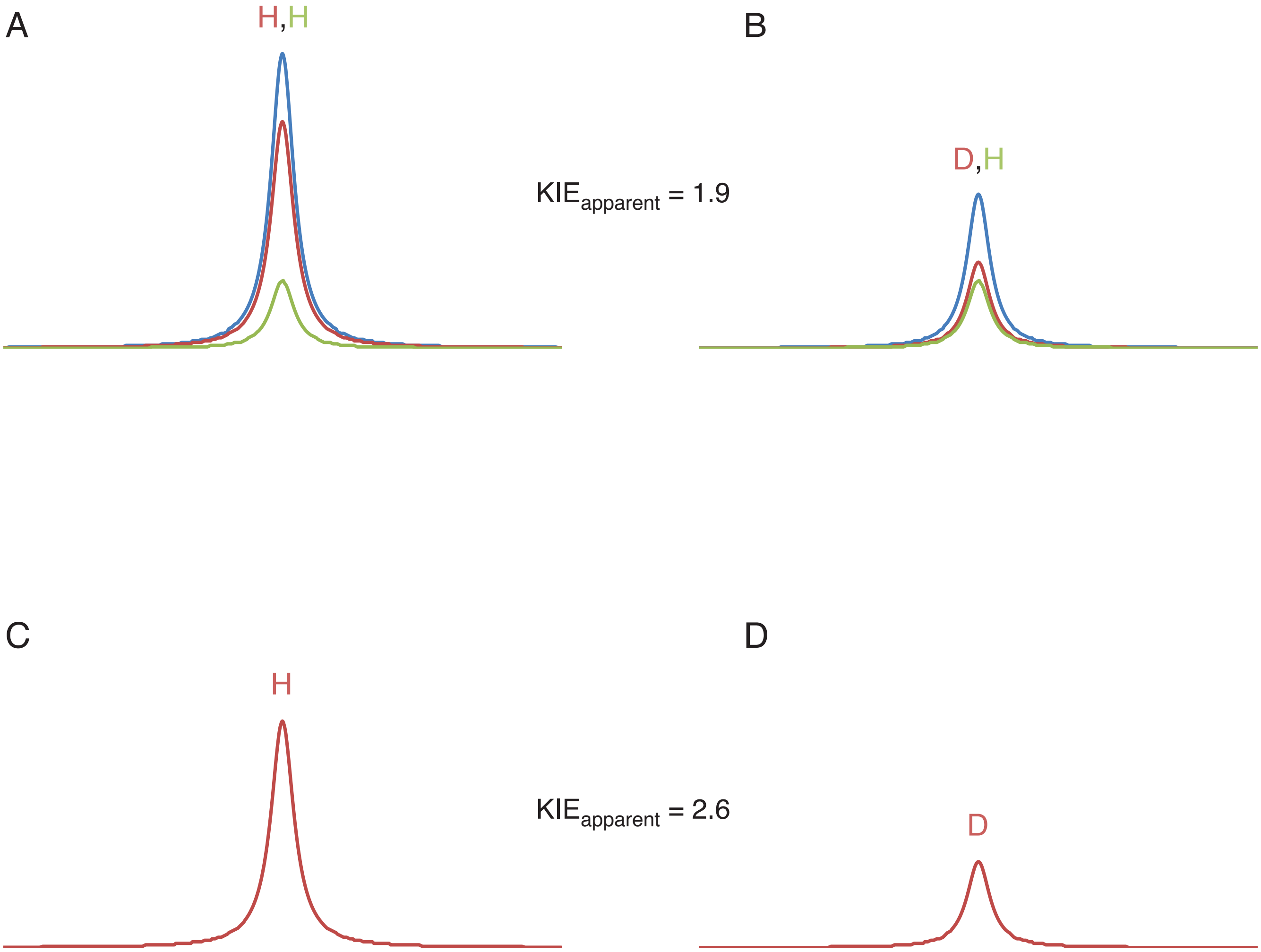

Figure S9

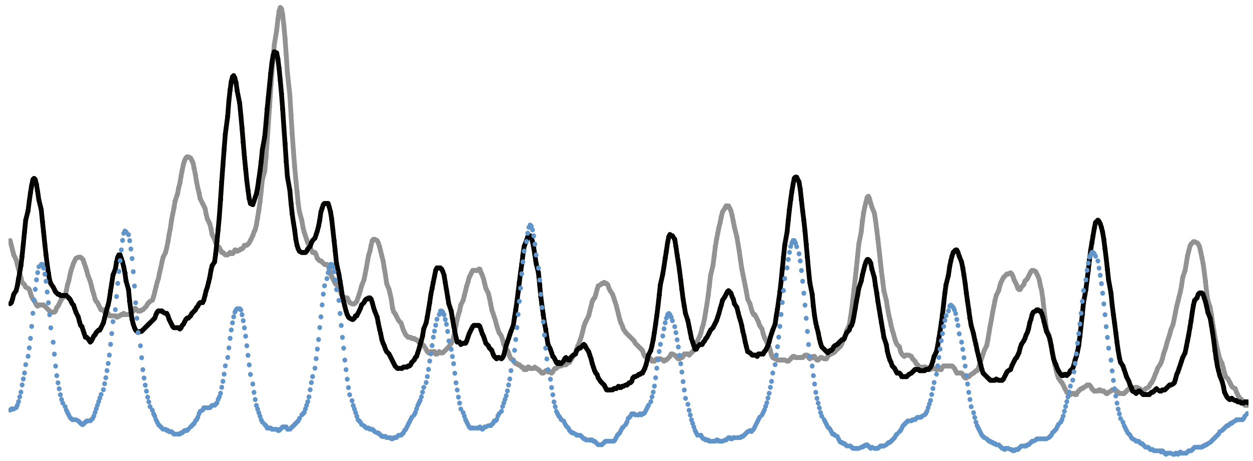

Figure S10

A

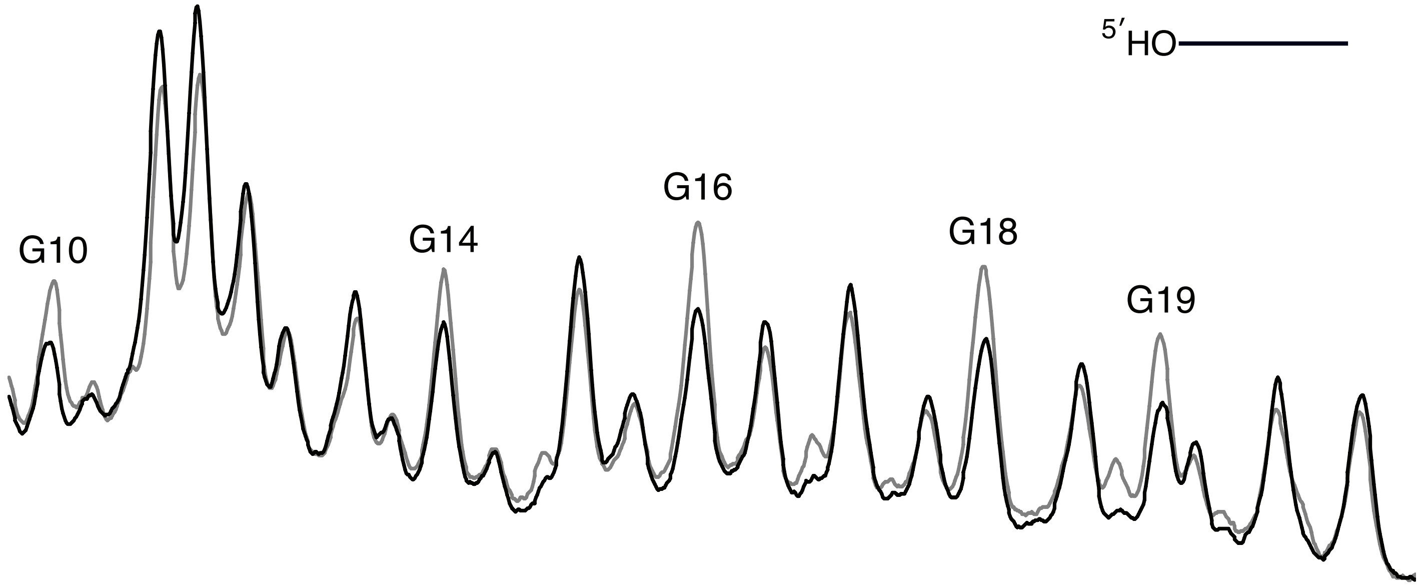

B

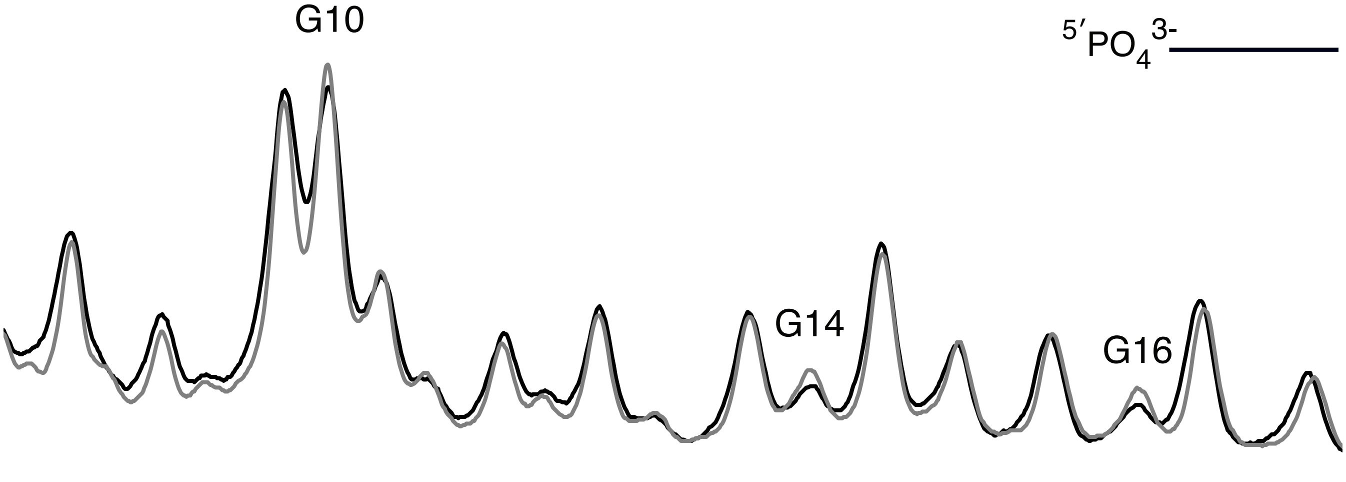

Figure S11

A

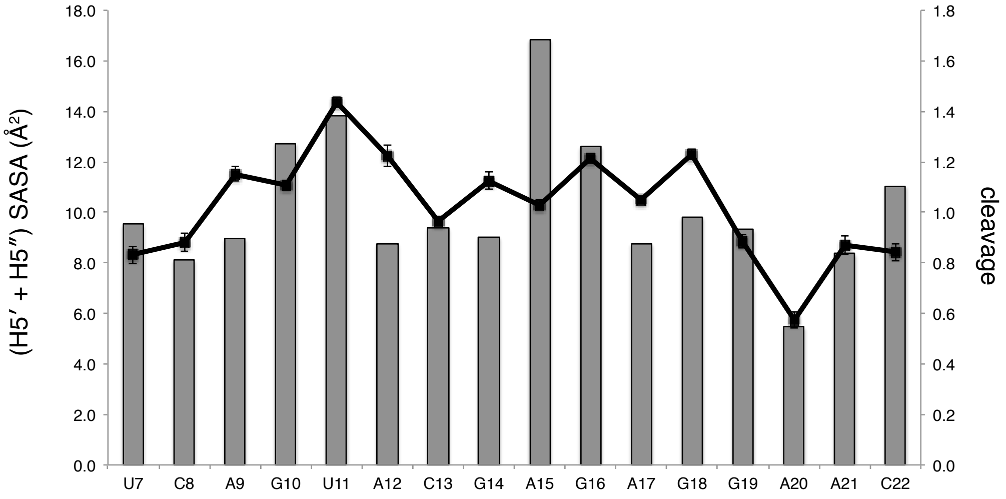

B

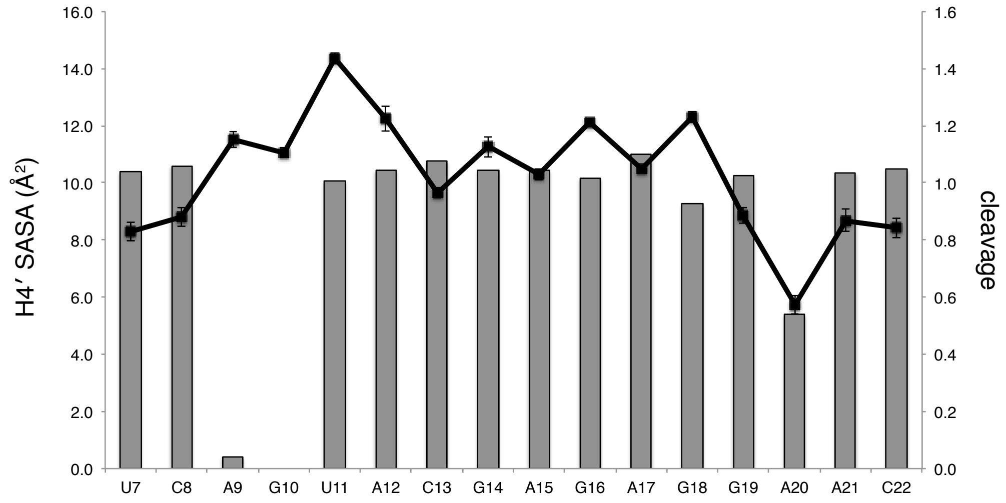

C

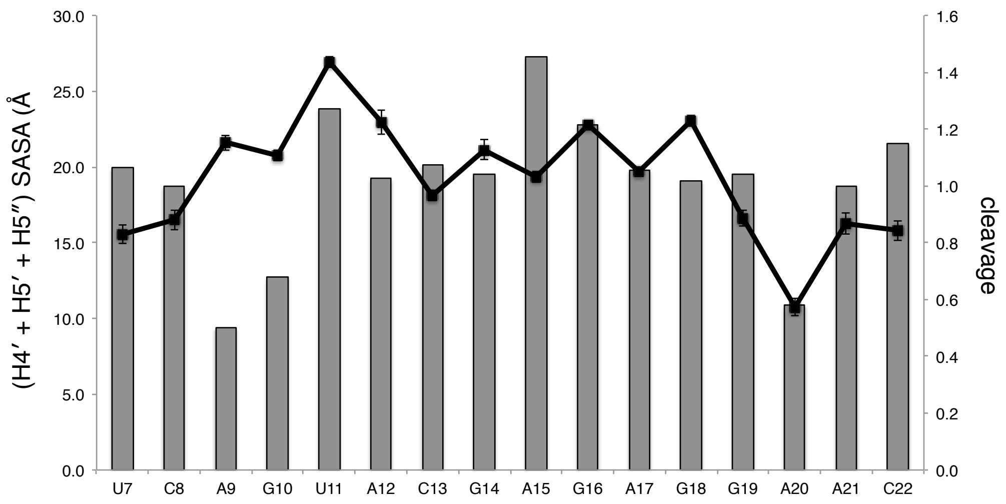

Figure S12

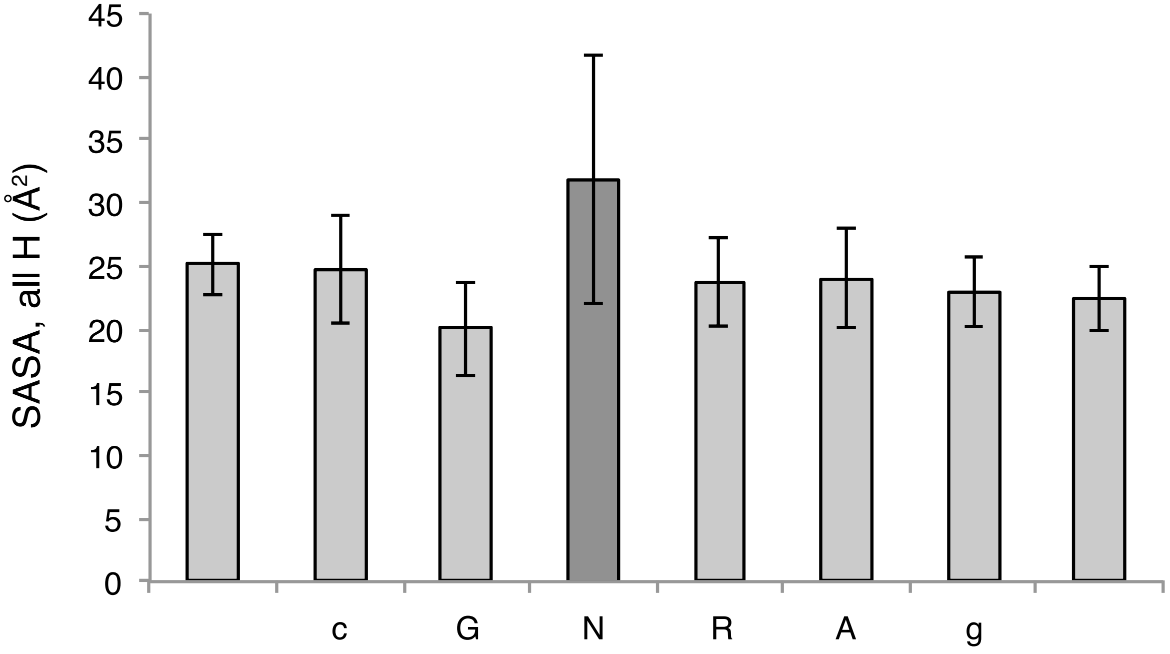

Supplement: SUPPLEMENTARY DATA [file supp_gku934_nar-01930-f-2014-File008.pdf]
